# *Liebetanzomycespolymorphus* gen. et sp. nov., a new anaerobic fungus (Neocallimastigomycota) isolated from the rumen of a goat

**DOI:** 10.3897/mycokeys.40.28337

**Published:** 2018-10-10

**Authors:** Akshay Joshi, Vikram B. Lanjekar, Prashant K. Dhakephalkar, Tony M. Callaghan, Gareth W. Griffith, Sumit Singh Dagar

**Affiliations:** 1 Bioenergy Group, Agharkar Research Institute, Pune, India Agharkar Research Institute Pune India; 2 Institute of Biological, Environmental and Rural Sciences, Cledwyn Building, Aberystwyth University, Aberystwyth, SY23 3DD, Wales, UK Aberystwyth University Aberystwyth United Kingdom; 3 Commercial Mushroom producers Co-Operative Society Ltd., Units 7 & 8, Newgrove Industrial Estate, Ballinode Road, Monaghan, Ireland Commercial Mushroom producers Co-Operative Society Ltd. Monaghan Ireland

**Keywords:** Anaerobe, fungal diversity, novel genus, phylogeny, rumen fungi, taxonomy

## Abstract

An extended incubation strategy to culture slow growing members of anaerobic fungi resulted in the isolation of a novel anaerobic fungus from the rumen of a goat after 15 days. The novel genus, represented by type strain G1SC, showed filamentous monocentric thallus development and produced uniflagellate zoospores, hence, showing morphological similarity to the genera *Piromyces*, *Buwchfawromyces*, *Oontomyces* and *Pecoramyces*. However, strain G1SC showed genetic similarity to the genus *Anaeromyces*, which, though produces uniflagellate zoospore, also exhibits polycentric thallus development. Moreover, unlike *Anaeromyces*, strain G1SC did not show hyphal constrictions, instead produced a branched, determinate and anucleate rhizoidal system. This fungus also displayed extensive sporangial variations, both exogenous and endogenous type of development, short and long sporangiophores and produced septate sporangia. G1SC utilised various complex and simple substrates, including rice straw and wheat straw and produced H_2_, CO_2_, formate, acetate, lactate, succinate and ethanol. Phylogenetic analysis, using internal transcribed spacer 1 (ITS1) and D1/D2 domain of large-subunit (LSU) rRNA locus, clearly showed a separate lineage for this strain, near *Anaeromyces*. The ITS1 based geographical distribution studies indicated detection of environmental sequences similar (93–96%) to this strain from cattle faeces. Based on morphological and molecular characterisation results of strain G1SC, we propose a novel anaerobic fungus *Liebetanzomycespolymorphus***gen. et sp. nov.**, in the phylum *Neocallimastigomycota*.

## Introduction

Anaerobic fungi inhabit the gut of various herbivorous animals where they play a pivotal role in the degradation of lignocellulosic feed (Gruninger et al. 2014). Some recent reports even suggest the presence of anaerobic fungi outside the gut of terrestrial animals and within the gut of sea animals and sediments (Ivarsson et al. 2016; Edwards et al. 2017; Picard 2017). Taxonomically, anaerobic fungi are the only member of phylum *Neocallimastigomycota*, class *Neocallimastigomycetes*, order *Neocallimastigales* and family *Neocallimastigaceae* (Hibbett et al. 2007). So far, ten genera of these fungi have been reported, namely *Anaeromyces*, *Caecomyces*, *Cyllamyces*, *Neocallimastix*, *Orpinomyces*, *Piromyces*, *Buwchfawromyces*, *Feramyces*, *Oontomyces* and *Pecoramyces*, the last four of which have been described very recently (Callaghan et al. 2015; Dagar et al. 2015; Hanafy et al. 2017; Hanafy et al. 2018).

The total number of anaerobic fungal species is reported to be 29, but this number is not validated due to several taxonomy related issues, including incorrect or repetitive naming (Paul et al. 2018). Several culture-independent studies, moreover, suggest the presence of at least 25 genus- and 119 species-equivalent taxa of anaerobic fungi, highlighting the need for continuous attempts for the cultivation of these additional taxa into axenic cultures (Kittelmann et al. 2012; Koetschan et al. 2014; Paul et al. 2018). This suggested number of total genera and species may even be higher as some of the primers used during routine ITS-based studies are also known to neglect certain cultures (Callaghan et al. 2015). These observations, therefore, reaffirm the need to look into vastly unexplored and unreported diversity of anaerobic fungi.

Many of the anaerobic fungal genera like *Orpinomyces*, *Caecomyces*, *Neocallimastix*, *Piromyces* etc. have a ubiquitous occurrence and can be found in diverse animal species, while some are reportedly host-specific (Paul et al. 2018). The genus *Oontomyces* has been reported to be a camel-specific genus (Dagar et al. 2015) and some uncultured genus designates have been reported to be specific to hosts like Somali ass, wallaby or American bison (Paul et al. 2018). Similarly, the presence of recently described genera *Pecoramyces* and *Feramyces* has been found limited to foregut fermenters and wild undomesticated animals, respectively (Hanafy et al. 2018). However, it is interesting to note that many genera remain uncultured even from the routinely sampled domesticated herbivores, a fact which needs further attention. In this paper, we report the isolation of a novel anaerobic fungus *Liebetanzomycespolymorphus*, isolated from goat rumen after prolonged incubation of 15 days.

## Materials and methods

### Sampling, cultivation and preservation

The rumen digesta samples were collected from goats (n=3) slaughtered at Kondhwa slaughterhouse, Pune (India). The samples were immediately brought to the laboratory (within 1 h), pooled and homogenised for 10 minutes under the gas phase of CO_2_ before making dilutions (up to 10^-4^) in the anaerobic diluent (McSweeney et al. 2005). The serum roll bottle method (Miller and Wolin 1974) was used to isolate pure cultures of anaerobic fungi from different dilutions. Briefly, the inoculum (0.5 ml) was added to 125 ml glass serum bottles containing 10 ml fungal culture medium (pH 6.8 ± 0.1), which comprised (per litre) of 3 g yeast extract, 5 g tryptone, 150 ml each of solution 1 (0.3% K_2_HPO_4_), solution 2 [0.3% KH_2_PO_4_, 0.6% (NH_4_)_2_SO_4_, 0.6% NaCl, 0.06% MgSO_4_.7H_2_O and 0.06% CaCl_2_.2H_2_O] and clarified rumen fluid, 1 ml resazurin (0.1%), 1 ml hemin (0.05%), 6 g NaHCO_3_, 1 g L-cysteine-HCl, 5 g cellobiose and 20 g agar. The antibiotics, benzylpenicillin and streptomycin sulphate (final concentration 200 µg/ml), were also added to inhibit bacterial growth.

All the roll bottles were incubated at 39±1 °C for 3 weeks and inspected regularly for the development of fungal colonies. The morphologically distinct colonies were picked under anaerobic conditions and inoculated into the fresh liquid culture medium. The serum roll bottle method was repeated two more times to get the axenic fungal cultures. All the cultures were cryopreserved at -80 °C and -196 °C for short-term and long-term storage, respectively using ethylene glycol (final concentration 0.64 M) as the cryoprotectant (Callaghan et al. 2015).

## Morphological characterisation

The colony morphology of the cultures was measured after 3 d growth on cellobiose roll bottles, using a stereomicroscope (Leica M205 FA) equipped with a digital camera (Leica DFC450 C). For documenting the microscopic features, the cultures were grown on different carbon sources like rice straw, wheat straw, cellulose, xylan, starch, cellobiose, lactose, maltose, sucrose, glucose, xylose and fructose for 3 d. The microscopic features like thallus morphology, shape and size of sporangia, zoospore shape and flagellation etc. were documented using a differential interference contrast (DIC) microscope (Olympus BX53) equipped with a digital camera (Olympus DP 73) and scanning electron microscope (Carl Zeiss EVO MA15), respectively. The samples for scanning electron microscopy were prepared as described by Ho et al. (1988). To determine the monocentric/polycentric growth patterns, nuclei positions were visualised following straining with bisbenzimide (Fliegerova et al. 2002) or DAPI (Callaghan et al. 2015) using a fluorescence microscope (Nikon Eclipse 80i) equipped with a monochrome digital camera (Media Cybernetics) or confocal microscope (Leica TCS SP8), respectively. All images were processed in GIMP (version 2.8.14) and then compiled using Inkscape (version 0.91) software.

### Molecular characterisation and phylogenetic analysis

For molecular characterisation, the genomic DNA was extracted using the CTAB DNA extraction protocol (Sirohi et al. 2013). The complete internal transcribed spacer (ITS1-5.8S-ITS2; ITS) and D1/D2 domain of large-subunit (LSU) ribosomal DNA were amplified using ITS1/ITS4 and NL1/NL4 primer pairs (Edwards et al. 2017). The sequencing was outsourced to 1^st^ BASE (Singapore) and the obtained sequences were compiled and edited manually using BioEdit software (Hall 1999). Since most of the culture-independent studies have generated only ITS1 sequences, the ITS sequence was trimmed to obtain only the partial ITS1 region. Sequence similarity searches were performed using GenBank BLASTn. For phylogenetic analyses, the ITS1 and LSU sequences representing different anaerobic fungal genera and uncultured representatives, were downloaded from the NCBI GenBank database. All the sequences were aligned using the ClustalW programme (Thompson et al. 1994) with default settings in MEGA7 (Kumar et al. 2016). The aligned sequences were used to construct a phylogenetic tree in MEGA7 using the maximum-likelihood method based on the Tamura-Nei model (Tamura and Nei 1993) and tested by 500 bootstrap replications. The genus *Gromochytriummamkaevae* was used as the outgroup for both ITS1 (accession number: KF586842) as well as LSU (accession number: NR_132054) based trees. The ITS1 and LSU alignments have been submitted to TreeBASE under submission ID 22988.

### Substrate utilisation, enzyme activities and fermentation product analyses

The obtained strains were grown in a fungal culture medium without yeast extract or tryptone and cellobiose was replaced by different substrates (Table 1) as a carbon source (Hanafy et al. 2017; Hanafy et al. 2018). Following initial growth for 2 d on monosaccharides and disaccharides and 5 d on polysaccharides, the cultures were subcultured three times at 10% inoculum size to evaluate their substrate utilisation abilities. The growth was measured in terms of visible biomass accumulation and total gas production. The cultures were scored based on their ability to grow luxuriantly (++) or slowly (+) within the stipulated incubation periods or following extended incubation up to 10 days. The avicelase, CMCase, xylanase and β-glucosidase activities of type strain were also determined following its growth on cellulose, xylan, wheat straw and rice straw for 5 d as per the method described previously (Dagar et al. 2018). The fermentation gases (H_2_, CO_2_), volatile fatty acids (VFAs; acetate, propionate, butyrate etc.) and alcohols (ethanol and butanol) of growth positive substrates were determined using gas chromatographs equipped with flame ionisation or thermal conductivity detectors, similar to previous reports (Dighe et al. 1998; Kamalaskar et al. 2010; Singh et al. 2016). The organic acids (formate, lactate, succinate, malate etc.) were analysed using HPLC LC20A (Shimadzu, Japan) equipped with a refractive index detector (Thakker et al. 2006). An ordination of the obtained fermentation products was also generated to see if there is any pattern of substrate utilisation using Non-metric Multidimensional Scaling (NMDS) analysis based on the distance matrix in PAST 3.20 software (Hammer et al. 2001).

**Table 1.** Substrate utilisation pattern of *Liebetanzomycespolymorphus* strains G1SC and G6SC compared with other genera of monocentric and uniflagellate filamentous anaerobic fungi.

**Substrate**		**Lp**	**Am**	**Pc**	**Pr**
Polysaccharide	Rice straw	++	ND	ND	ND
	Wheat straw	++	ND	ND	ND
	Cellulose	++	++	++	++
	Xylan	++	++	++	++
	Starch	++	++	++	++
	Inulin	–	–	–	++
	Raffinose	–	–	++	++
	Chitin	–	ND	ND	–
	Alginate	–	ND	ND	–
	Pectin	++	–	–	–
Disaccharide	Cellobiose	++	++	++	++
	Sucrose	+	++	++	++
	Maltose	++	++	++	++
	Trehalose	–	ND	ND	+
	Lactose	+	+	++	–
Monosaccharide	Glucose	++	++	++	++
	Xylose	++	++	++	++
	Mannose	–	–	–	++
	Fructose	++	++	++	++
	Arabinose	–	–	–	–
	Ribose	–	ND	–	–
	Glucuronic acid	–	ND	ND	–
	Galactose	–	–	–	–
Peptide	Peptone	–	ND	ND	–
	Tryptone	–	ND	ND	–

Strains: **Lp***Liebetanzomycespolymorphus* strain G1SC and G6SC **Am***Anaeromycesmucronatus* strain BF2 (Breton et al. 1990) **Pc***Piromycescommunis* strain SM5 (Trinci et al. 1994) **Pr***Pecoramycesruminantium* strain C1A and S4B (Hanafy et al. 2017). – (negative), ++ (positive), + (weak growth/ slow growth/ growth visible after 4–10 d of incubation), ND (not documented)

## Results and discussion

### Growth and morphological characterisation

For the routine anaerobic fungal isolation, the inoculated roll tubes are usually incubated for 2–4 d after which the developed colonies are picked anaerobically and pure cultures are obtained. However, we chose to incubate the roll tubes for an extended period of time i.e. up to 3 weeks. The decision for extended incubation was taken in the wake of the fact that several genera of anaerobic fungi remain to be uncultured and incubation time may be one of the limiting factors. As shorter incubation times favours fast-growing cultures, some slow growing cultures might be omitted and which may grow after prolonged incubation. The prolonged incubation might also help some cultures to cope better in stressed conditions of different growth environment, the presence of antibiotics or some oxygen exposure during sample collection or processing. In a previous study, prolonged incubation was recommended for the isolation of anaerobic bacteria from clinical specimens and correct bacteriological diagnosis (Wren 1980). Janssen et al. (2002) also speculated that the extended incubation period may help isolation of previously uncultured groups of soil bacteria. In this case, the extended incubation of 15 d resulted in the development of two fungal colonies, but no further growth even after 21 d of incubation. Following isolation and establishment of pure cultures, the morphological, molecular and substrate utilisation characteristics of both strains, namely G1SC and G6SC were found to be identical. Therefore, one of them, i.e. strain G1SC, was denoted as the type strain and used for detailed characterisation. The type strain has been deposited to the MACS collection of microorganisms (MCM), Agharkar Research Institute, Pune, India under the accession number MCMB-1469.

**Figure 1.**
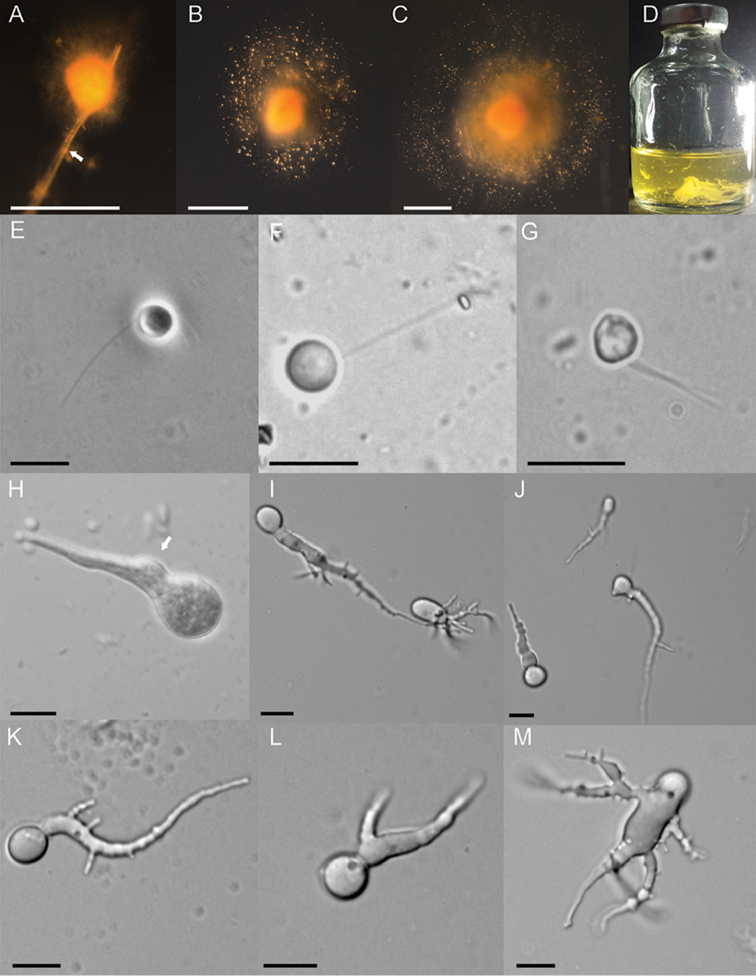
Macroscopic and microscopic features of *Liebetanzomycespolymorphus*. Colony morphology on agar roll tubes (**A–C**), showing the development of a colony attached (**A**) to a straw particle (arrowed), dense growth in the centre surrounded by numerous sporangia and zoospores (**B–C**) causing expansion of colony size. Growth in liquid medium showing a biofilm-like growth (**D**). Zoospores are spherical and Uniflagellate (**E–F**) or biflagellate (**G**). Germinating zoospore (**H**) showing a zoospore cyst (arrowed), presence and absence of sporangiophore indicating the endogenous and exogenous type of sporangial development (**I**) and different shapes of sporangia (**I, J**). Early stages of thallus development showing a single (**K**), bifurcated (**L**) and multifurcated (**M**) rhizoidal system. Scale bar: 1 mm (**A–C**); 10 µM (**E–M**).

The colony morphology of strain G1SC is shown in Fig. 1A–C, showing 1–2 mm sized colony, attached to rice straw (Fig. 1A) and at different stages of growth. A large number of newly formed sporangia at the periphery of the colony were observed, as shown by numerous dot-like structures of varying sizes (Fig. 1B–C). A similar observation was also made in broth culture, where numerous fungal thalli, attached to the bottom of glass bottles, were seen on the initial period of growth (Suppl. material 1: Figure S1), which later developed into a thin mat or biofilm-like structure (Fig. 1D) similar to *Pecoramyces* (Hanafy et al. 2017). Zoospores were abundantly produced, mostly uniflagellate (Fig. 1E–F) and rarely biflagellate (Fig. 1G), spherical to ovoid in size (5–6 µm in diameter) and the flagellum 15–20 µm in length. The zoospores were found to germinate either endogenously or exogenously (Fig. 1H–I) into different shapes of sporangia (Fig. 1J) like globose or ellipsoidal at the very early stages of development. Different forms of rhizoidal development, like long single rhizoid (Fig. 1K), two rhizoids (Fig. 1L) and even multiple rhizoids originating from sporangia (Fig. 1M), were also seen.

**Figure 2.**
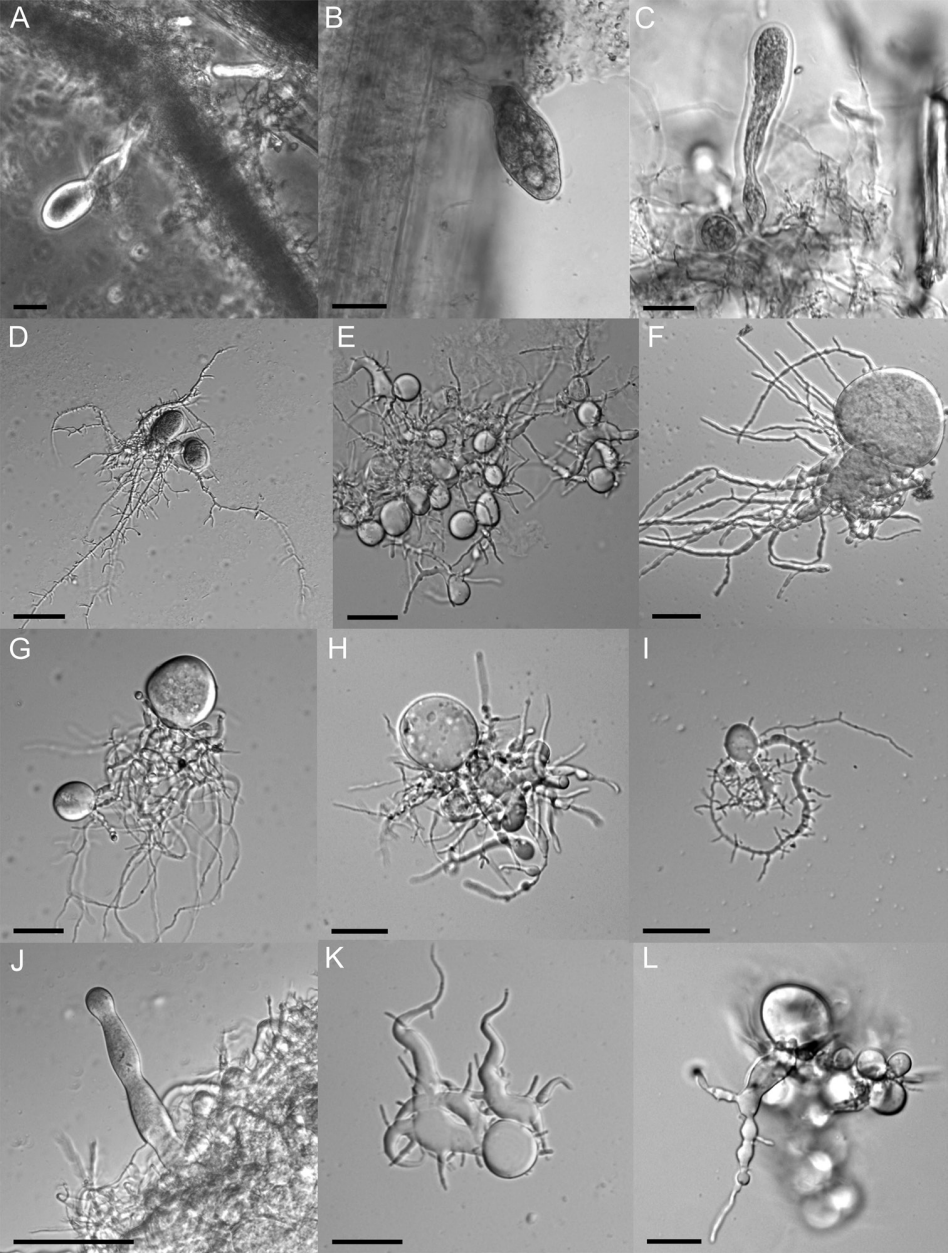
Microscopic features of *Liebetanzomycespolymorphus* showing pleomorphism in sporangial and rhizoidal structures, when grown on different carbon sources like rice straw (**A**), wheat straw (**B**), cellulose (**C**), xylan (**D**), starch (**E**), cellobiose (**F**), lactose (**G**), maltose (**H**), sucrose (**I**), glucose (**J**), xylose (**K**) and fructose (**L**). The exogenous sporangia appearing on a sporangiophore (**A–B**) and endogenous sporangia of different shapes (**C–L**) are shown. Scale bar: 20 µM.

**Figure 3.**
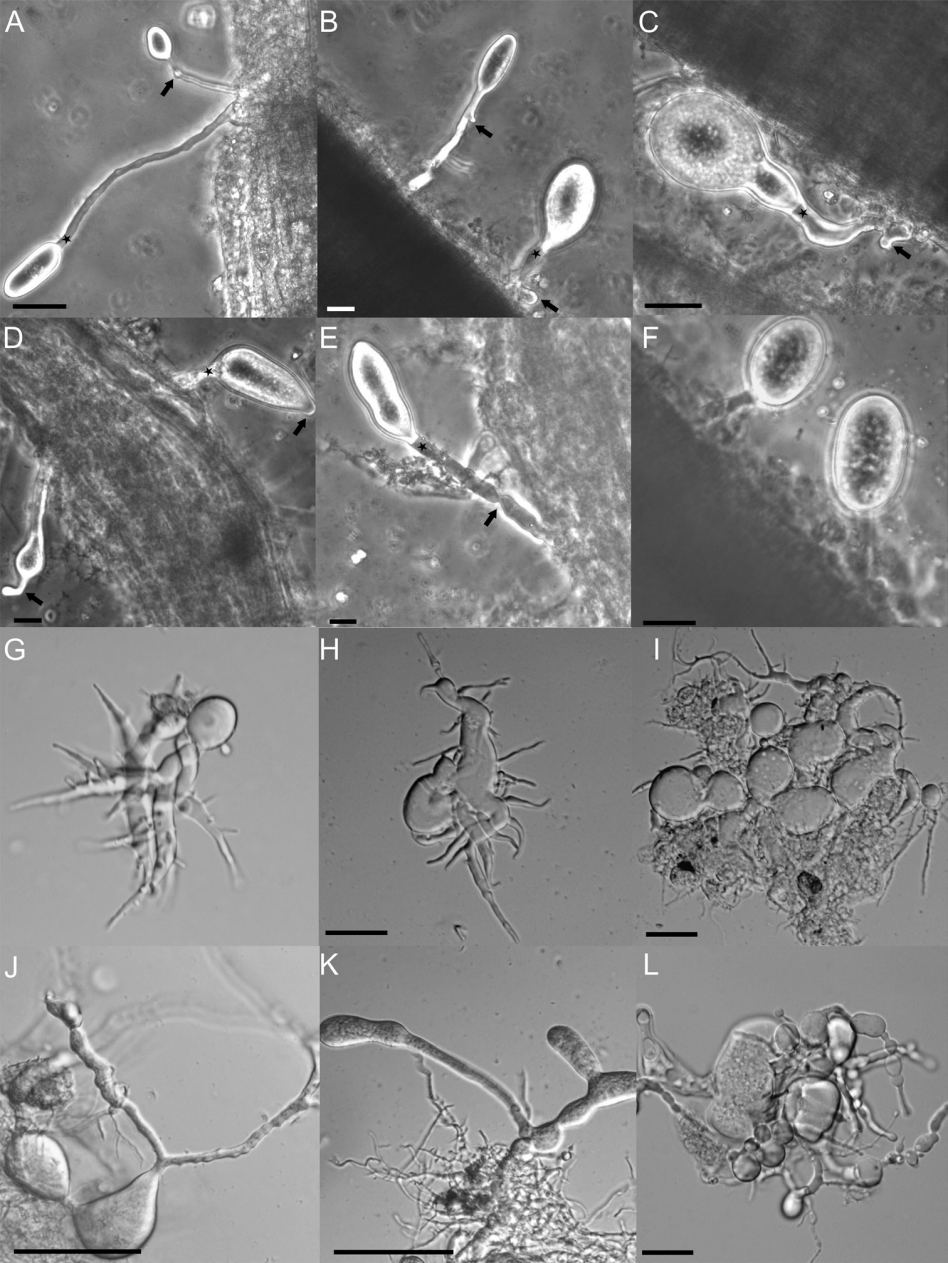
Microscopic images of *Liebetanzomycespolymorphus*. On rice straw (**A–F**) sporangia of varying sizes and shapes, like elongate (**A**), ellipsoid (**B**), ovoid (**C**), clavate (**E**) and globose (**F**). The zoospore cyst (**A–C** arrowed) is also visible, highlighting bipolar germination. Sporangiophore of varying length and shape, from short to long (**A**), sometimes eggcup shaped (**C**) and constricted (**E** arrowed) is shown. The sporangia with a papilla (**D** arrowed) and septum (**B, C, D, E** starred) are also indicated. Sporangia and thalli of irregular morphology on xylose (**G–I**) and cellobiose (**J–L**), including some pseudo-intercalary sporangia (**J–L**) can be observed. Scale bar: 20 µM.

We also noticed pleomorphism in sporangial and rhizoidal structures of strain G1SC on different substrates like rice straw (Fig. 2A), wheat straw (Fig. 2B), cellulose (Fig. 2C), xylan (Fig. 2D), starch (Fig. 2E), cellobiose (Fig. 2F), lactose (Fig. 2G), maltose (Fig. 2H), sucrose (Fig. 2I), glucose (Fig. 2J), xylose (Fig. 2K) and fructose (Fig. 2L). In the case of complex substrates like rice straw, wheat straw, cellulose and xylan, the rhizoidal growth was observed to be more extensively branched. Conversely, thicker and less branched rhizoidal growth was noticed on dimeric and monomeric substrates, probably due to the ready availability of fermentable sugars. It was also observed that, on rice and wheat straw, an exogenous type of sporangia development with short or long sporangiophores was more prominent in comparison to endogenous sporangia on all other substrates. The presence of sporangiophores on complex straw particles might be helpful for sporangia to come out of straw and release zoospores to further areas for faster colonisation. The pleomorphism, in morphological features with a change in culture conditions, particularly with carbon source, is also well described earlier (Brookman et al. 2000; Gruninger et al. 2014). However, the sporangial shapes varied, not only on different carbon sources, but also on the same carbon source (Fig. 3). Different sporangial shapes like globose, ellipsoid, clavate, ovoid, with or without a papilla were noticed on the same substrate i.e. rice straw as shown in Fig. 3A–F. Similar polymorphism was also observed on several other substrates, e.g. xylose (Fig. 3G–I) and cellobiose (Fig. 3J–L), where conspicuous irregular sporangial structures were seen. These findings, therefore, highlight the pleomorphic nature of strain G1SC, irrespective of the carbon sources used in the growth medium.

**Figure 4.**
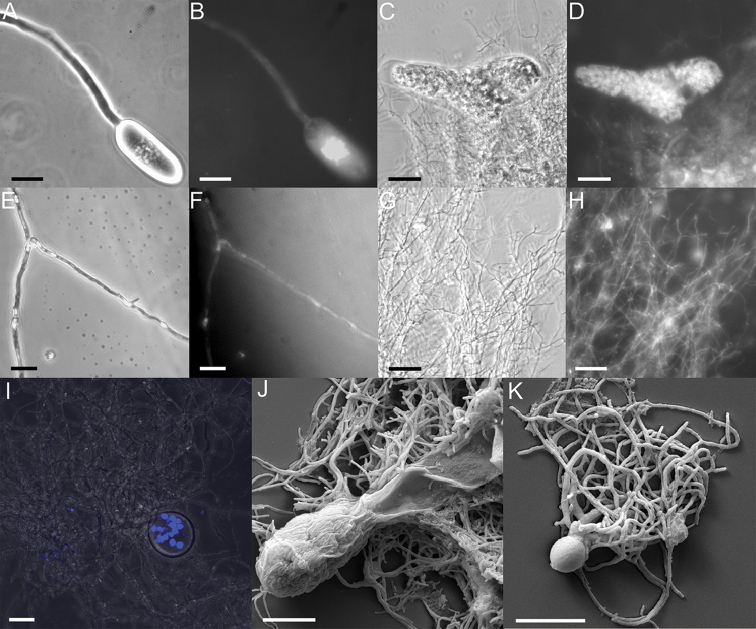
Phase contrast (**A, C, E, G**), fluorescence (**B, D, F, H**) and confocal (**I**) microscopic images of *Liebetanzomycespolymorphus* showing elongate (**A–B**) and triangular (**C–D**) sporangium filled with zoospores. Nuclei were seen in sporangium but not in sporangiophore (**B**) or rhizoidal system (**F, H, I**). No constrictions were observed in rhizoids. Scanning electron microscopy images of elongate (**J**) and globose (**K**) sporangium showing monocentric thallus. Scale bar: 20 µM (**A–I**); 10 µM (**J–K**).

The sporangial size also varied in diameter (10–90 µm wide, 10–75 µm long), always borne at the terminal end of a variable length sporangiophore (15–80 µm), in the case of exogenous development. Several cyst-like structures were also visible on the sporangiophore (Fig. 3A–C) highlighting bipolar germination of zoospores. In some cases, an eggcup shaped sporangiophore at the bottom of sporangium (Fig. 3C) and a constricted sporangiophore were also noted (Fig. 3E). In the case of soluble substrates, few pseudo-intercalary sporangia, similar to *Oontomyces* and *Feramyces* (Dagar et al. 2015; Hanafy et al. 2018), were also noted (Fig. 3J–L). The thallus development was clearly monocentric as evident by the mature sporangia full of zoospores (Fig. 4A–D, I), anucleated rhizoids (Fig. 4E–I) and a single thallus having a single sporangium (Fig. 4J–K). All these morphological features were similar to various *Piromyces* spp., *Buwchfawromyces, Oontomyces* and *Pecoramyces*, which also have the monocentric thallus and extensive variations in sporangial shape and size and produce uniflagellate zoospores (Ho and Barr 1995). Similar to *P.rhizinflatus*, the sporangia of strain G1SC showed a constricted, isthmus-like neck (Fig. 2C, J), sporangiophore appearing like an eggcup (Fig. 3C) like in *P.communis* and sporangium with a papilla (Fig. 3D) similar to *P.mae* (Ho and Barr 1995; Li et al. 1990).

The similarities in morphological features of monocentric and uniflagellate genera make it very difficult to identify and differentiate this group of anaerobic fungi. It is also interesting to note that, so far, the maximum number of species has been described in the *Piromyces* genus and most of the newly described genera of anaerobic fungi share morphological similarities with these different species, including strain G1SC. These observations point towards the possibility that some of these newly described genera might have been isolated previously as well but were identified as different species of *Piromyces*. It is also worth noting that all newly described genera have primarily been described using molecular tools, thus emphasising the need to use these modern tools.

### Molecular characterisation and phylogenetic analysis

The successful amplification of ITS and LSU regions yielded product sizes of ca. 700 bp and 750 bp, respectively. The obtained sequences were submitted to NCBI GenBank to obtain the accession numbers for strain G1SC (MH468765 and MH468763; ITS and LSU) and G6SC (MH468766 and MH468764; ITS and LSU). The ITS region based sequence similarity search results showed that strain G1SC was 88.14% and 87.03% similar to *A.robustus* (accession number: NR 148182) and *A.contortus* (accession number: MG605706), respectively. Likewise, the LSU region of strain G1SC was most closely similar (97.71%) to *A.contortus* (accession number: MG605690). Although the sequence similarity of ca. 97% usually relates to a novel culture at the species level with its nearest match, the stark morphological dissimilarities of strain G1SC with *A.contortus* led to its classification as a novel genus.

**Figure 5.**
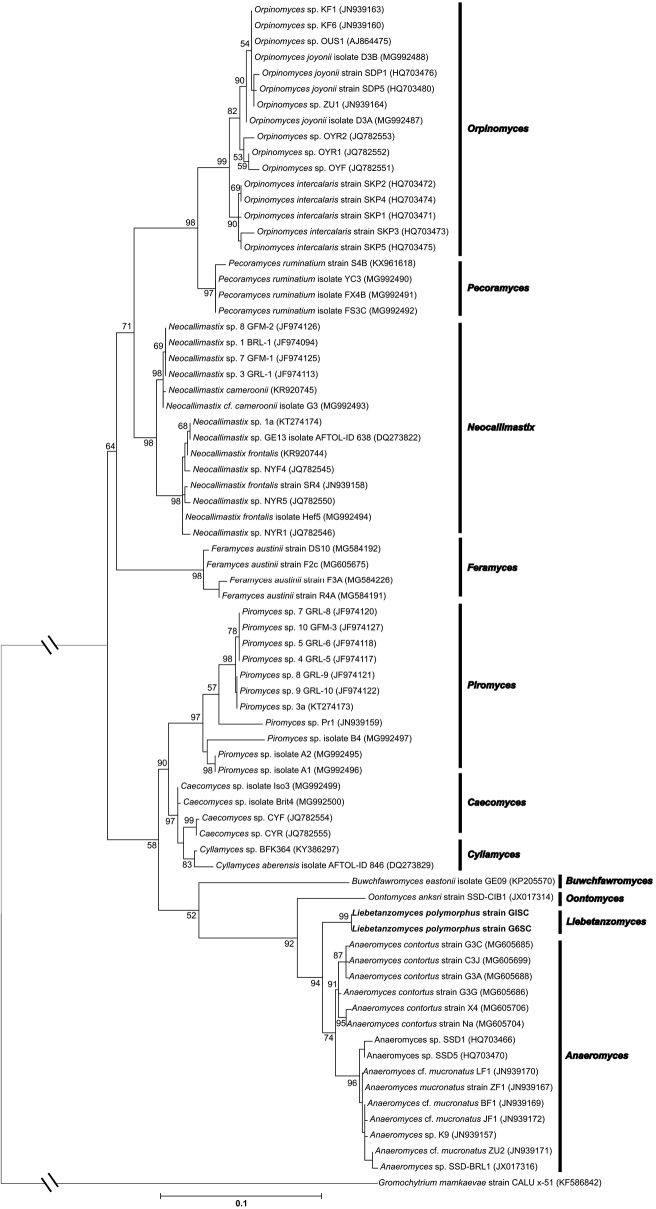
The LSU based maximum-likelihood tree is showing the phylogenetic position of *Liebetanzomycespolymorphus* with other members of phylum *Neocallimastigomycota*. Bootstrap values (>50%) based on 500 replicates are indicated at branching points. The GenBank accession number of each strain is listed in parentheses. Scale bar: 0.1 substitutions per site.

**Figure 6.**
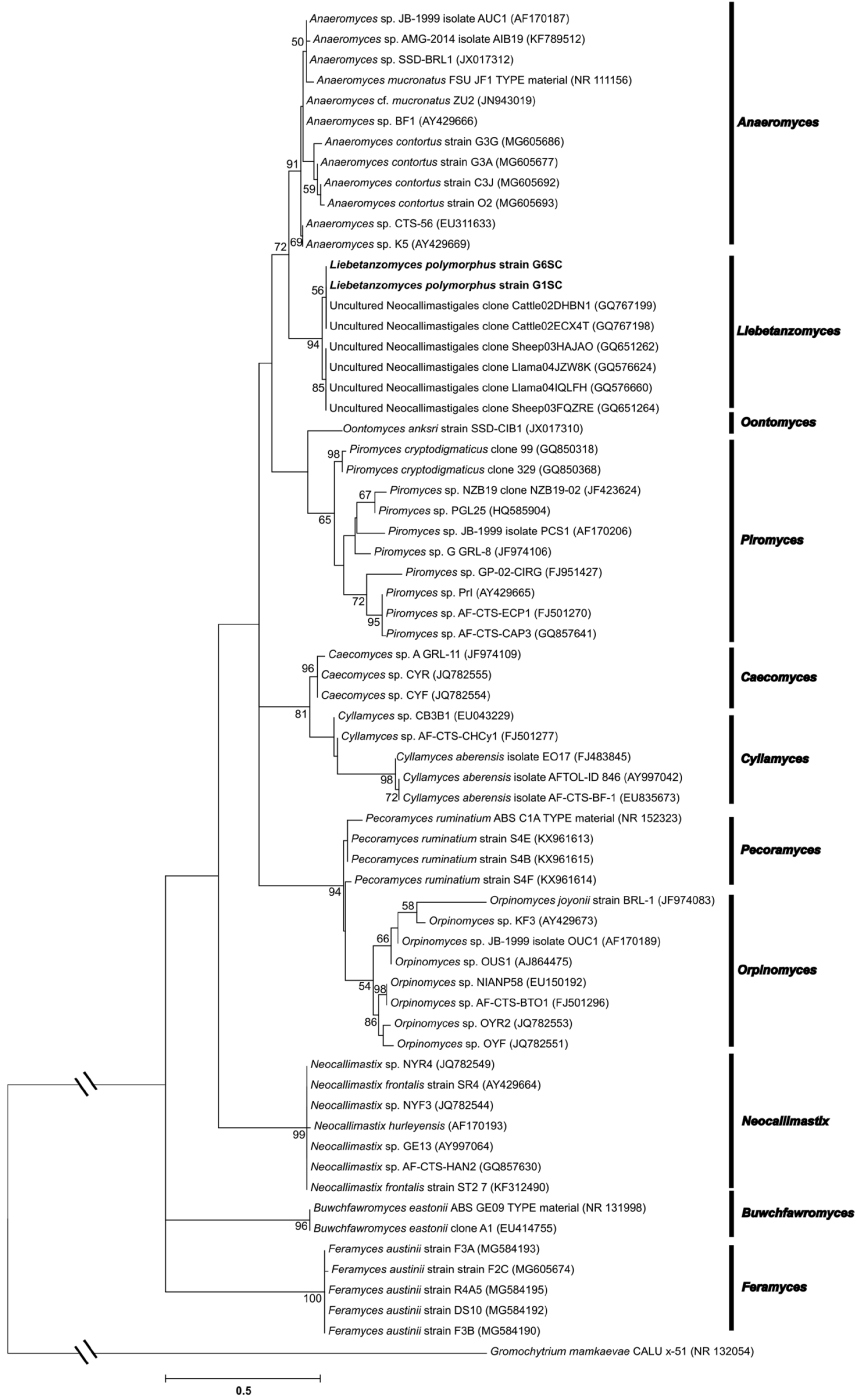
The ITS1 based maximum-likelihood tree is showing the phylogenetic position of *Liebetanzomycespolymorphus* with nearest uncultured clones and other members of phylum *Neocallimastigomycota*. Bootstrap values (>50%) based on 500 replicates are indicated at branching points. The GenBank accession number of each strain is listed in parentheses. Scale bar: 0.5 substitutions per site.

To better understand the ecological distribution of strain G1SC, only ITS1 based searches were performed, which indicated <96% sequence similarities with several uncultured representatives of phylum Neocallimastigomycota (Suppl. material 2: Figure S2) reported from the USA (Liggenstoffer et al. 2010). The nearest matches were with different clones obtained from cattle (93–96%) and other clones of cattle, sheep and llama (88%-92%), indicating the presence of members of the G1SC clade in cattle and close relatives in sheep and llama. The nearest matches of G1SC clade were also found in the SP4 clade designated by Paul et al. (2018), represented by accession number GQ767184, which consisted of 120 sequences of uncultured fungi, all from cattle faeces. The results, hence, indicate the presence of related fungi in both large as well as small ruminants. Surprisingly, no similar sequences were obtained from goat, which might be due to a lesser number of culture-independent studies conducted on these animals. The phylogenetic analysis of LSU and ITS1 regions showed distinct lineage for the G1SC clade, supported by high bootstrap values (Fig. 5 and Fig. 6), nearest to the Anaeromyces cluster. The results, therefore, underscore the importance of morphology-based identifications along with molecular tools, as none on its own can be relied on for accurate identification of anaerobic fungi.

### Substrate utilisation, enzyme activities and fermentation product analyses

The substrate utilisation profiles of strains G1SC and G6SC exhibited exactly identical patterns (Table 1). Both these strains could utilise various polymeric and monomeric substrates. Our results were also compared to substrate utilisation profiles of other genera of monocentric and uniflagellate filamentous anaerobic fungi. All these genera were found to utilise cellulose, xylan, starch, cellobiose, sucrose, maltose, glucose and fructose, while none utilised chitin, ribose, peptone and tryptone as the sole carbon and energy source. For inulin, raffinose, alginate, pectin, trehalose, mannose, arabinose, glucuronic acid and galactose, varying results were obtained. Interestingly, only strains G1SC and G6SC were found positive for pectin utilisation amongst compared genera.

The enzyme activities (µmol/ml/h) of type strain G1SC showed maximum enzyme activities on crude substrates like rice straw and wheat straw, instead of pure cellulose and xylan (Table 2). The highest avicelase (1.05 ± 0.19) and xylanase (88.33 ± 1.00) activities were obtained on rice straw, while highest CMCase (7.73 ± 0.31) and β-glucosidase (1.64 ± 0.08) activities were measured in wheat straw. When compared to the enzyme activities of *Neocallimastix*, *Piromyces*, *Orpinomyces* and *Anaeromyces*, grown on wheat straw (Dagar et al. 2018), the strain G1SC displayed poor β-glucosidase activities. The avicelase activities of strain G1SC were comparable to *Orpinomyces* and *Anaeromyces*, but lower than most strains of *Neocallimastix* and *Piromyces*. The CMCase and xylanase activities of strain G1SC were comparable to *Orpinomyces*, *Neocallimastix* and most strains of *Piromyces*, but higher than *Anaeromyces*. These results suggest that strain G1SC is a moderate lignocellulose degrader and can be of use in applications involving lignocellulose degradation.

**Table 2.** Enzyme activities (µmol/mL/h) of *Liebetanzomycespolymorphus* strain G1SC on different substrates at 5 d of incubation.

**Enzyme activity**	**Substrate used**			
	**Rice straw**	**Wheat straw**	**Cellulose**	**Xylan**
Avicelase	1.05 ± 0.19	0.68 ± 0.10	0.73 ± 0.06	–
CMCase	4.55 ± 0.14	7.73 ± 0.31	5.56 ± 0.98	–
Xylanase	88.33 ± 1.00	79.44 ± 0.11	–	54.69 ± 2.31
Β-glucosidase	1.53 ± 0.07	1.64 ± 0.08	0.69 ± 0.02	

– ±: standard deviation of three replicates.

The substrate utilisation results were also confirmed by measuring the fermentation products on each substrate (Table 3). The strain G1SC produced hydrogen, carbon dioxide, formate, acetate, lactate, succinate and ethanol on all substrates, but did not produce propionate, butyrate, butanol or malate which is in line with the previous reports (Trinci et al. 1994; Edwards et al. 2017). The absence of propionate and butyrate also implies that there was no bacterial contamination and all products are solely of fungal origin. The fermentation product quantities also indicated the efficiency of substrate utilisation. The substrates like sucrose and lactose, which recorded poor growth after 2 d of incubation, also showed lesser production of formate, acetate and lactate and the absence of succinate. Likewise, the polymeric substrate like rice straw, wheat straw, cellulose and xylan showed lesser production of hydrogen, formate, acetate, lactate, succinate and ethanol at 2 d of incubation (Table 3), which increased substantially after 5 d (Suppl. material 4: Table S1). The NMDS plot showed clear clustering of various carbon sources based on fermentation products, an observation duly supported by a low stress value (Suppl. material 3: Figure S3). These results, therefore, signify the usefulness of metabolite profiling for measuring the substrate utilisation abilities of anaerobic fungi.

**Table 3.** Fermentation products of *Liebetanzomycespolymorphus* strain G1SC on different substrates after 2 d of incubation.

**Substrate**	**Fermentation product (in mM)**						
	**Hydrogen**	**Carbon dioxide**	**Formate**	**Acetate**	**Lactate**	**Succinate**	**Ethanol**
Rice straw	58.10 ± 3.57	4.82 ± 0.16	16.43 ± 0.04	26.07 ± 0.24	1.79 ± 0.04	0.29 ± 0.00	10.35 ± 0.05
Wheat straw	45.42 ± 2.98	5.46 ± 0.13	11.44 ± 0.07	19.77 ± 0.44	1.72 ± 0.04	0.24 ± 0.00	8.27 ± 0.13
Cellulose	26.86 ± 3.11	6.38 ± 0.05	4.61 ± 0.05	13.26 ± 0.52	0.73 ± 0.02	ND	7.27 ± 0.01
Xylan	27.71 ± 2.48	7.12 ± 0.11	5.01 ± 0.06	8.30 ± 0.12	4.01 ± 0.06	0.12 ± 0.00	6.96 ± 0.03
Starch	44.09 ± 0.54	5.99 ± 0.03	9.45 ± 0.10	14.58 ± 0.28	15.11 ± 0.38	0.34 ± 0.00	7.69 ± 0.13
Pectin	42.59 ± 1.18	6.38 ± 0.09	7.64 ± 0.08	11.32 ± 0.22	3.56 ± 0.04	0.15 ± 0.01	8.01 ± 0.12
Cellobiose	58.05 ± 0.63	5.64 ± 0.03	9.75 ± 0.02	15.69 ± 0.29	9.35 ± 0.04	0.30 ± 0.01	8.46 ± 0.04
Sucrose	29.21 ± 1.02	6.99 ± 0.05	3.77 ± 0.10	7.61 ± 0.44	0.66 ± 0.00	ND	9.11 ± 0.06
Maltose	53.26 ± 3.01	5.59 ± 0.12	8.26 ± 0.13	17.45 ± 0.40	10.16 ± 0.06	0.22 ± 0.00	7.85 ± 0.05
Lactose	39.08 ± 1.83	6.51 ± 0.08	4.26 ± 0.01	8.98 ± 0.13	0.47 ± 0.01	ND	8.52 ± 0.04
Glucose	49.79 ± 0.86	5.84 ± 0.04	10.11 ± 0.18	16.75 ± 0.19	7.18 ± 0.02	0.31 ± 0.00	8.65 ± 0.09
Xylose	53.80 ± 1.41	5.62 ± 0.09	8.38 ± 0.19	12.58 ± 0.47	7.90 ± 0.06	0.16 ± 0.00	8.39 ± 0.03
Fructose	61.05 ± 1.59	5.28 ± 0.06	11.79 ± 0.05	17.05 ± 0.71	15.97 ± 0.03	0.30 ± 0.00	9.10 ± 0.05

– ±: standard deviation of three replicates.

#### Liebetanzomyces polymorphus

Taxon classificationFungiNeocallimastigalesNeocallimastigaceae

Joshi, G.W. Griff. & Dagar, gen. et sp. nov.

##### Diagnosis.

Strictly anaerobic fungus with determinate, monocentric thallus with single terminal sporangium of varying shape and size and uniflagellate zoospores. The clade is defined by the sequences accession numbers MH468765 (ITS1, 5.8S, ITS2 complete) and MH468763 (LSU, partial sequence). The most genetically similar genus is *Anaeromyces*, which is defined as forming a polycentric thallus (Breton et al. 1990.FEMS Microbiol. Lett. 58, p.177), in contrast to the monocentric *Liebetanzomyces*.

A strict anaerobic fungus, isolated from the rumen of a goat. In roll tubes of cellobiose agar medium, the fungus forms medium to large circular colonies (0.5–2 mm diam.). In liquid cellobiose medium, the fungal thalli attach to the sides and bottom of glass bottles. Monocentric thallus development, producing a single terminal sporangium per thallus. Also, the fungus produces few pseudo-intercalary sporangia but only on soluble substrates. An extensive anucleate rhizoidal system without constrictions is formed. The sporangia vary in size (10–90 µm wide, 10–75 µm long), shape (globose, ellipsoid, clavate, ovoid, elongate or irregular) and sometimes bear papillae. The sporangium is borne on variable length sporangiophore (15–80 µm), sometimes forming an eggcup-like structure below the sporangium or showing cyst-like structure. Zoospores are produced abundantly, mostly uniflagellate, rarely biflagellate, spherical (5–6 µm in diameter) in size and flagellum of 15–20 µm in length. The zoospores may germinate either endogenously or exogenously. The clade is defined by the sequences accession numbers MH468765 (ITS1, 5.8S, ITS2 complete) and MH468763 (LSU, partial sequence). The ex-type culture (strain G1SC) is stored cryogenically in liquid nitrogen at bioenergy group, Agharkar Research Institute, Pune, India. The holotype is a 3 d old culture of G1SC preserved in 5% glutaraldehyde and deposited at the MACS-collection of microorganisms (MCM) of Agharkar Research Institute, Pune, India and isotype material at the Aberystwyth University biorepository (code ABS).

##### Etymology.

‘*Liebetanz*’ is assigned to honour Erwin Liebetanz (Liebetanz 1910) by taking all nine letters of his surname (i.e. LIEBETANZ) and who was the first to observe anaerobic gut fungus *Piromyces* as a flagellated organism in 1910; myces = the Greek name for fungus.

The specific epithet *polymorphus* is for different polymorphic sporangial shapes displayed by this fungus.

## Conclusions

The morphological and molecular characterisation results clearly indicate that the strain G1SC represents a novel genus *Liebetanzomycespolymorphus* in the phylum *Neocallimastigomycota*. *Liebetanzomycespolymorphus* displays a monocentric thallus and produces uniflagellate zoospores, thus, it is morphologically similar to *Piromyces, Buwchfawromyces*, *Oontomyces* and *Pecoramyces*. *Liebetanzomycespolymorphus* exhibits extensive sporangial variations and is genetically near but dissimilar to *Anaeromyces*. The ITS1 and LSU based phylogenetic analysis also confirmed the distinct lineage of *Liebetanzomycespolymorphus*. The results suggest that both morphological and molecular tools should be used in tandem to determine the uniqueness of any anaerobic fungal culture, as the use of either one independently can result in a wrong identification.

## Supplementary Material

XML Treatment for Liebetanzomyces polymorphus

## Supplementary materials

Supplementary material 1 Figure S1 Data type: multimedia Explanation note: Photographs showing fungal thalli (marked by arrows) of *Liebetanzomyces polymorphus* attached to the sides (A) and bottom (B) of glass bottles when grown in liquid medium. This is an open access article distributed under the terms of the Creative Commons Attribution License (CC BY 4.0), which permits unrestricted use, distribution, and reproduction in any medium, provided the original author and source are credited.Akshay Joshi, Vikram B. Lanjekar, Prashant K. Dhakephalkar, Tony M. Callaghan, Gareth W. Griffith, Sumit Singh Dagar

Supplementary material 2 Figure S2 Data type: multimedia Explanation note: The ITS1 based maximum-likelihood tree based on the Tamura 3-parameter model (Tamura 1992) is showing the phylogenetic position of *Liebetanzomyces polymorphus* with nearest uncultured clones. The genus *Anaeromyces mucronatus* (accession number: NR_111156) was used as the outgroup. Bootstrap values (>50%) based on 500 replicates are indicated at branching points. The GenBank accession number of each strain is listed before the name. Bar, 0.02 substitutions per site. This is an open access article distributed under the terms of the Creative Commons Attribution License (CC BY 4.0), which permits unrestricted use, distribution, and reproduction in any medium, provided the original author and source are credited.Akshay Joshi, Vikram B. Lanjekar, Prashant K. Dhakephalkar, Tony M. Callaghan, Gareth W. Griffith, Sumit Singh Dagar

Supplementary material 3 Figure S3 Data type: multimedia Explanation note: The Non-metric multidimensional scaling (NMDS) plot showing clustering of various carbon sources based on fermentation products. Stress value, 0.012. This is an open access article distributed under the terms of the Creative Commons Attribution License (CC BY 4.0), which permits unrestricted use, distribution, and reproduction in any medium, provided the original author and source are credited.Akshay Joshi, Vikram B. Lanjekar, Prashant K. Dhakephalkar, Tony M. Callaghan, Gareth W. Griffith, Sumit Singh Dagar

Supplementary material 4 Table S1 Data type: species data Explanation note: Fermentation products of *Liebetanzomyces polymorphus* strain G1SC on different substrates after 5 d of incubation. This is an open access article distributed under the terms of the Creative Commons Attribution License (CC BY 4.0), which permits unrestricted use, distribution, and reproduction in any medium, provided the original author and source are credited.Akshay Joshi, Vikram B. Lanjekar, Prashant K. Dhakephalkar, Tony M. Callaghan, Gareth W. Griffith, Sumit Singh Dagar
