# Supplementary figures and images for: Liebetanzomycespolymorphus gen. et sp. nov., a new anaerobic fungus (Neocallimastigomycota) isolated from the rumen of a goat
Source: MycoKeys. 2018 Oct 10;(40):89–110. doi: 10.3897/mycokeys.40.28337 (PMC6198248; doi:10.3897/mycokeys.40.28337)

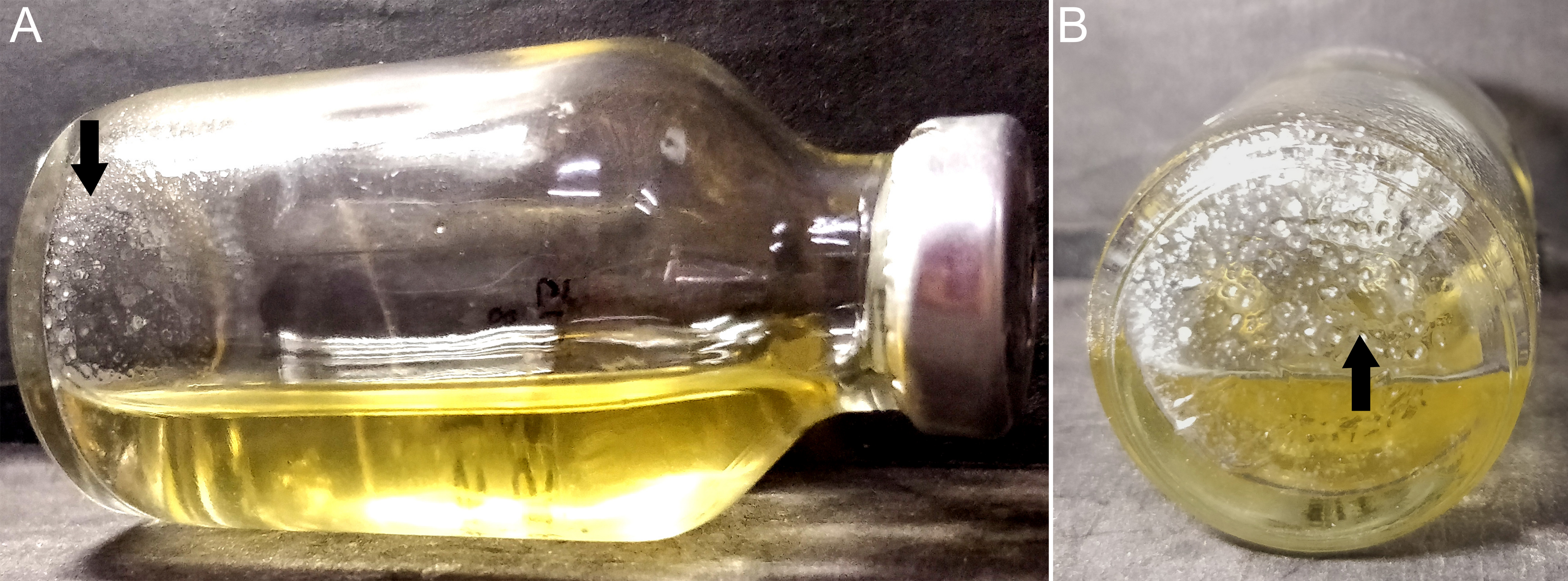

Supplement: Supplementary material 1 — Figure S1 [file mycokeys-40-089-s001.png]

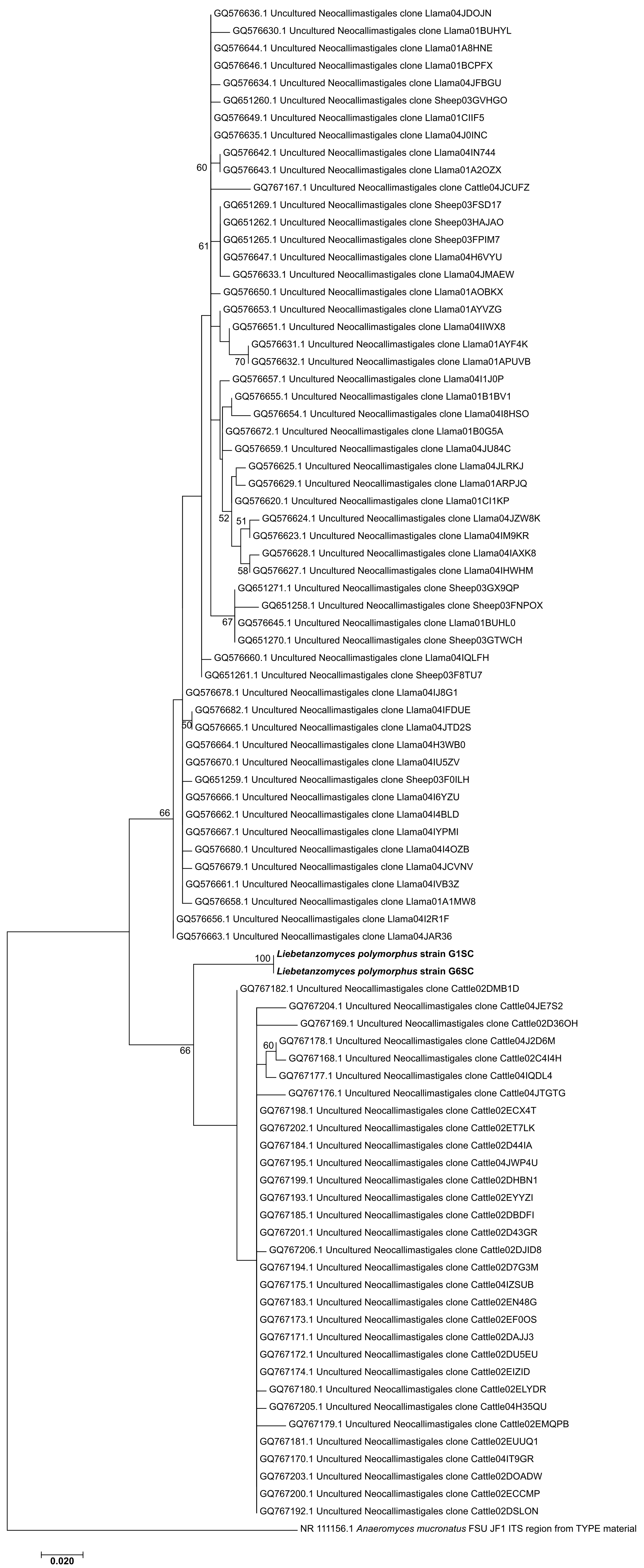

Supplement: Supplementary material 2 — Figure S2 [file mycokeys-40-089-s002.png]

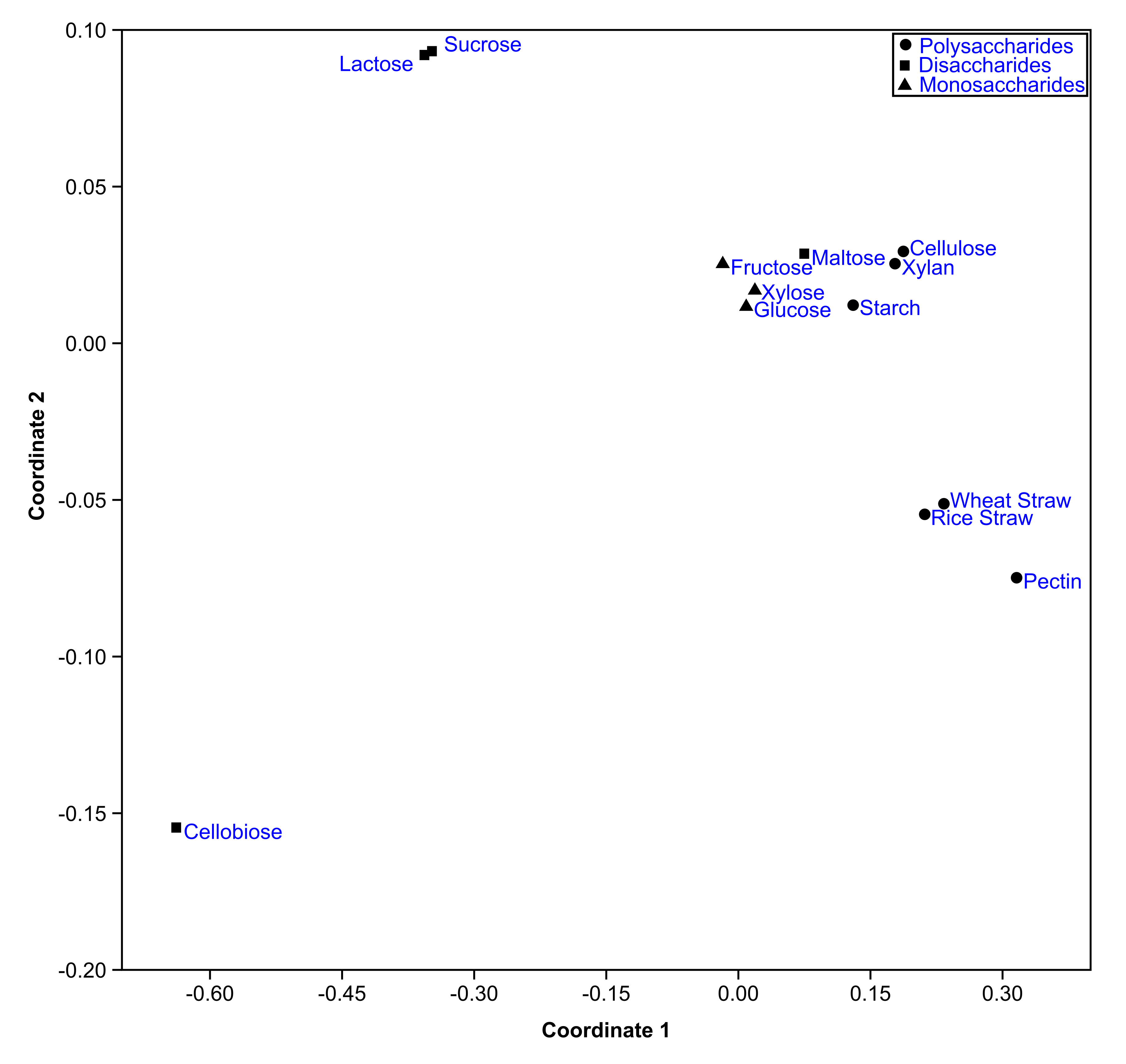

Supplement: Supplementary material 3 — Figure S3 [file mycokeys-40-089-s003.png]
