# Supplementary material for: Liebetanzomycespolymorphus gen. et sp. nov., a new anaerobic fungus (Neocallimastigomycota) isolated from the rumen of a goat
Source: MycoKeys. 2018 Oct 10;(40):89–110. doi: 10.3897/mycokeys.40.28337 (PMC6198248; doi:10.3897/mycokeys.40.28337)
Supplement: Supplementary material 4 — Table S1 [file mycokeys-40-089-s004.docx]

**Supplementary Table 1** Fermentation products of *Liebetanzomyces polymorphus* strain G1SC on different substrates after 5 d of incubation

| **Substrate** | **Fermentation product (in mM)** | | | | | | |
| --- | --- | --- | --- | --- | --- | --- | --- |
|  | Hydrogen | Carbon dioxide | Formate | Acetate | Lactate | Succinate | Ethanol |
| Rice straw | 65.01±1.74 | 3.88±0.05 | 29.42±1.59 | 29.64±0.22 | 8.27±0.68 | 0.91±0.05 | 21.67±0.27 |
| Wheat straw | 69.57±0.27 | 4.32±0.02 | 18.13±0.08 | 31.05±0.63 | 4.96±0.05 | 0.70±0.03 | 23.99±0.54 |
| Cellulose | 46.36±1.47 | 6.24±0.07 | 11.43±0.15 | 15.71±0.23 | 5.73±0.27 | 0.43±0.01 | 20.25±0.52 |
| Xylan | 50.97±2.88 | 4.56±0.14 | 17.44±0.35 | 11.05±0.47 | 24.69±0.17 | 0.68±0.02 | 23.91±0.12 |

±: standard deviation of three replicates
